# Tidy Surroundings

**Published:** 1874-10

**Authors:** 


### TIDY SURROUNDINGS.

Every one who has traveled through our country will remember the pleasurable sensations experienced in passing by a farm-house where everything seems to have been freshly painted and whitewashed; these things add greatly to the attractiveness and the saleability of all country places, to say nothing of the greater bodily health and moral purity promoted by such surroundings. A good housekeeper will be known anywhere along Fifth Avenue or Thirty-Fourth Street by the appearance of the "risers" of the steps which lead to their street doors. Some look fresh and clean, others are covered with green mould and streaked with dust, suggestive of bayous and frog ponds. The easiest method of removing fungus and moss and mould from stone or wood is to moisten freely with petroleum, turning to a brown or black, and soon falling off, leaving the surfaces nice and clean. Paint for wood, enduring and preservative, as well as economical, can be made thus: Dissolve a pound of blue vitriol in a gallon of water, wash the wood with it by spreading it on with a brush; then spread on another wash made by dissolving half a pound of yellow prussiate of potash in a gallon of water; this forms a coating of brown color, which protects the wood from the weather and the depredation of insects; made more durable in freshness if a coating of linseed oil is spread over with a brush.

A beautiful and lasting whitewash for fences and outhouses is made thus: Dissolve water-lime cement in milk, mix it to a paint consistency and give three or four coats with a brush. By renewing the application once in two years, buildings will look fresh all the time at a trifling cost.

For rough, outside walls, exposed to the weather, there is nothing better than fresh lime, slaked with simple water. If the wall is hard and smooth, add as much fine sand as the mixture can take up and can be applied with a brush.

For inside walls a quarter of a pound of glue dissolved in warm water and stirred into five gallons of the wash will make it adhere to the walls better. The white of eggs instead of glue, in the same proportions, adds to the whiteness. But for common fencing and outhouses use the following, spread with a whitewash brush: "Take a clean, water-tight barrel or other suitable cask, and put into it half a bushel of lime. Slack it by pouring water over it, boiling hot, and in sufficient quantity to cover it five inches deep, and stir it briskly till thoroughly slacked. When the slacking has been effected, dissolve it in water, and add two pounds of sulphate of zinc, and one of common salt. These will cause the wash to harden, and prevent its cracking, which gives an unseemly appearance to the work. If desirable, a beautiful cream color may be communicated to above wash, by adding three pounds of yellow ochre, or a good pearl or lead color, by the addition of lamp, vine or ivory black. For fawn color, add four pounds umber—Turkish or American—(the latter is the cheapest), one pound Indian red, and one pound common lampblack. For common stone color, add four pounds raw umber, and two pounds lampblack." But one of the great drawbacks to farm houses, with all their paint and whitewash, and with all their mythical abundance of fresh eggs, *new* milk, luscious butter and vegetables just out of the ground, is the fly pest; they wake you up in the morning, they drop into the gravy at noon, and are lying around dead everywhere, or crawl over every eatable thing, thus leaving us to the second table. A fly-paper, which is not poisonous, is thus made: put one pound of quassia wood in five pints or pounds of water, boil it down to a quart; after it has stood all night in a warm place, strain the mixture; take the wood, add two pints of fresh water and boil down to one pint; strain and mix the two; then add half a pound of sugar, stir it until thoroughly dissolved, dip into this soft blotting or newspaper, hang on a line to dry, then spread it out; the flies feed on it and die.

KITCHEN SLOPS.

In many cases the slops of the kitchen are thrown out at the back door; this is an unsuspected cause of sickness and death in many households; they should be conveyed to a sink fifty yards away, and the ashes made in the house fires should be sprinkled over them every morning, altogether making quite an amount of valuable manure every few months.

